# An approach leveraging radiomics and model checking for the automatic early diagnosis of adhesive capsulitis

**DOI:** 10.1038/s41598-024-69392-6

**Published:** 2024-08-14

**Authors:** Giulia Varriano, Vittoria Nardone, Maria Chiara Brunese, Michela Bruno, Antonella Santone, Luca Brunese, Marcello Zappia

**Affiliations:** Department of Medicine and Surgery “V. Tiberio”, University of Molise, 86100 Campobasso, Italy

**Keywords:** Frozen shoulder, Radiomics, Medical imaging, Formal methods, Model checking, Computational biology and bioinformatics, Diseases, Medical research, Engineering, Computer science

## Abstract

Adhesive Capsulitis of the shoulder is a painful pathology limiting shoulder movements, commonly known as “Frozen Shoulder”. Since this pathology limits movement, it is important to make an early diagnosis. Diagnosing capsulitis relies on clinical assessment, although diagnostic imaging, such as Magnetic Resonance Imaging, can provide predictive or supportive information for specific characteristic signs. However, its diagnosis is not so simple nor so immediate, indeed it remains a difficult topic for many general radiologists and expert musculoskeletal radiologists. This study aims to investigate whether it is possible to use disease signs within a medical image to automatically diagnose Adhesive Capsulitis. To this purpose, we propose an automatic Model Checking-based approach to quickly diagnose the Adhesive Capsulitis taking as input the radiomic feature values from the medical images. Furthermore, we compare the performance achieved by our method with diagnostic results obtained by professional radiologists with different levels of experience. To the best of our knowledge, this is the first method for the automatic diagnosis of Adhesive Capsulitis of the Shoulder.

## Introduction

Adhesive capsulitis of the shoulder (ACS or AC) is a frequent pathology in the population, especially in the middle-aged population^1,2^. This pathology is protected by the limitation of shoulder movements, which is why it is called “frozen shoulder”^3^. Being a pathology that limits movement, it is important to make a timely diagnosis^4^.

The diagnosis of AC is mostly a clinical diagnosis, although diagnostic imaging may be predictive or helpful for some typical signs found in Magnetic Resonance Imaging (MRI)^4^. Among the typical signs for the non-contrast MRI and US (Ultrasound) are currently use in the diagnosis of shoulder pathologies. In particular, ultrasound are performing in the evaluation of the subcoracoid space^5^.

The synovial tissue of the glenohumeral joint devoid of capsular lining protrudes over the superior margin of the subscapularis muscle (subscapular recess) and progressively reduces the visibility of subcoracoid fat tissue due to chronic synovitis and local adhesions. This is particularly useful in the diagnosis of anterior shoulder pain and subcoracoid effusion^6,7^.

Ultrasound also allows to evaluate thickness coracohumeral ligament and thickness glenohumeral joint capsule; furthermore the last improvement of microvascular flow allow an earlier diagnosis of adhesive capsulitis of the shoulder^8^.

Concerning MRI diagnosis it correlates well with clinical findings. Radiological finding consists in thickening of the coracohumeral ligament and inferior joint capsule, edema of the axillary recess and rotator interval, and obliteration of the fat triangle inferior to the coracoid process^9,10^.

MRI plays an important role in excluding other pathologies causing shoulder pain, and to plan the best management. A correct clinical and radiological diagnosis of ACS is essential to establish correct therapy and restore the patient to full well-being^11^.

However, the diagnosis of capsulitis on MRI is challenging and it still remains a difficult topic for many general radiologists and expert musculoskeletal radiologists (MSK). As reported in the study by Zappia et al.^5^ and Sofka et al.^10^, not only the symptoms of AC can persist for more than 2 years, but, while the specificity of the MRI features is higher than 0.90, the sensitivity is still lower than 0.7. It has also been demonstrated that artificial intelligence is able and useful to improve the diffusion of high quality reports even in non specialized radiologists^12–14^.

The help of *Artificial Intelligence* (AI) and software capable of identifying the MRI signs of capsulitis, could be useful tools for clinicians, general radiologist, and also for expert MSK. Furthermore, several studies have demonstrated the presence of disease signs within the medical images to evaluate their diagnostic potential^15–17^. Thus, our aim is to improve the diagnostic sensitivity of MRI and improve the diagnostic accuracy of general radiologists.

Due to the above reasons, we want to investigate if it is possible to use the evidence of disease signs within a medical image to automatically detect the presence of AC. Precisely, we want to investigate if it is possible to develop automatic methods to diagnose AC based on medical image processing using Radiomics^18^. To classify AC presence in patients, we use the Model Checking technique which is a formal verification technique usually used to verify and test complex systems^19^. Precisely, Model Checking takes as input an abstract model that mimics the behavior of the system, and a property, i.e., a precise behavior that a system has to be met. In our specific case, the model is represented by a patient medical imaging exam and the property represents the disease signs of AC. Finally, we compare the effectiveness achieved by the proposed method with the performance reached with traditional diagnostic modalities, i.e., traditional diagnostics performed by radiologists having different levels of experience. A total of 11 doctors with several years of experience were recruited. In parallel, the doctors had to assess the presence or absence of AC in the same dataset used by the automatic method. These results were then compared with those provided by the automatic method in the Results Section.

To the best of our knowledge, this is the first study trying to develop a completely automatic approach aiding professionals to diagnose AC using the Model Checking technique.

## Theory

In the following, we provide some background information about Radiomics and Model Checking technique.

### Radiomics

Radiomics^20^ is an emerging field in medical imaging that involves the extraction and analysis of quantitative features from medical images, such as Computed Tomography (CT) scans, MRI, Positron Emission Tomography (PET) scans, and other types of images. These features are then analyzed using advanced computational and statistical methods to gain insights into tumor biology, prognosis, treatment response, and other clinical outcomes.

Radiomics is most commonly used in oncological and emergency disease, but it is also in muscoloskeleletal radiology^21–24^.

Furthermore, before features are extracted, medical images can be preprocessed to standardize and improve data quality. Additionally, to streamline analysis, techniques for Feature Selection and reduction are applied to identify the most informative and discriminative features. This selection helps reduce the dimensionality of the feature space and improve computational efficiency.

Thus, Radiomics allows to extract a large number of quantitative features (i.e., radiomic features) from medical images. These features can include measures of intensity, shape, texture, statistical properties, and other mathematical descriptors that capture various aspects of the tumor or Region of Interest (ROI). Radiomics has the potential to enhance clinical decision-making and contribute to the field of Precision Medicine by integrating non-invasive imaging data with clinical or other types of data about the patient.

### Model checking

Model checking^19^ is a formal verification technique used to verify whether a given system or model meets a specified set of properties or requirements. It is primarily used in Computer Science, Software Engineering, and hardware design to ensure the correctness of complex systems.

This technique is basically composed of three steps: (i) system model building, (ii) properties specification, and (iii) properties verification using a Model Checker tool. More precisely, the first step allows us to model the system as a finite automaton, i.e., a Labeled Transition System (LTS) reproducing the system behavior using states, transitions, and possible actions.

The second step consists of property specification using temporal logic. These properties represent requirements or behaviors that the system should satisfy. Temporal logic allows the specification of properties over time, such as “eventually”, “always”, “next”, etc. These properties can be safety properties (undesirable states are not reached) or liveness properties (desirable states are eventually reached).

Finally, the third step aims to verify behavioral properties against the system model using a Model Checker tool. The Model Checker systematically verifies whether the specified properties hold for all states in the state space. This is done by exhaustively traversing the state space and verifying the properties at each state. Roughly speaking, the Model Checker explores all possible states the system can reach during its execution where the whole state space is usually represented as a directed graph with nodes representing states and edges representing transitions.

In this paper, the Model Checker is used in the context of Formal Methods, i.e. mathematical techniques for the verification of complex systems. Thanks to special syntaxes, we are able to create mathematical models from radiomic features on which the Model Checker can act by checking the satisfaction of a requirement.

## Material and methods

The *goal* of this study is to early diagnose AC through medical image analysis. The *perspective* is to provide medical professionals with tools that can assist them in an early diagnosis of the disease and evaluate the effectiveness of the provided tools. The *context* of the study are 55 MRI scans belonging to 55 patients, out of which 32 are affected by AC and 23 are not.

At this point, we address the following two research questions (RQs):

${\textbf {RQ}}_1$ *Can AC be diagnosed using Model Checking and radiomic features?* We are interested in studying if it is possible to diagnose AC using automatic methodology. More precisely, we want to investigate whether radiomic features can be used to model a patient and can be used as a vector to detect the presence of AC in medical images. This research question aims to develop an automatic approach that extracts radiomic features from MRI and uses these features to create a formal model, then this model is used to verify if a patient is affected by AC or not using the Model Checking technique and formal properties.

${\textbf {RQ}}_2$ *To what extent does the proposed approach for AC diagnosis perform in terms of accuracy when compared to traditional diagnostic modalities?* We want to evaluate the effectiveness of the proposed tool when compared to traditional diagnostic modalities, i.e., traditional diagnostics performed by radiologists with different levels of experience. This research question aims to assess the effectiveness of our tool and identify possible usages.

We answer our research questions in three steps. First, we collected *Digital Imaging and Communications in Medicine* (DICOM) images from 55 patients, i.e., 32 affected by AC and 23 negatives. Second, we develop an automatic approach using radiomic features and Model Checking to automatically detect patients affected by AC. We also compute evaluation metrics to estimate the effectiveness of our approach in the categorization of patients into healthy and afflicted groups. Third, we ask radiologists to determine, using MRI images, whether patients were affected by AC or not and we compare the results with those obtained by our tool to assess its effectiveness and potential applications.

### Dataset

In this retrospective study, we used a dataset provided by Professor Marcello Zappia. This dataset consists of DICOM images from 55 patients who faced MRIs between 2022 and 2024. All MRI scans were carried out at the “Istituto Diagnostico Varelli SRL” and the Bioethics Committee of the University of Molise approved the study and waived the requirement for informed consent and the further need of guidelines (prot. no. 27789 of 12.06.2024). We enrolled 55 patients who arrived at our hospital with acute shoulder pain and limited movement, 32 were affected by AC and 23 were not.

Inclusion criteria:

- Shoulder pain and/or movement limitation started from 4-12 months (frozen phase);
- Signed consent to share data;
- Age > 18 years old.

Exclusion criteria:

- Other etiologies of shoulder pain;
- Other etiologies of limited shoulder movement;
- Denied consent to share data;
- Artifacts during MRI acquisition;
- Previous adhesive capsulitis.

Note that comorbidites, i.e., diabetes, cancer, or trauma, were not considered exclusion criteria, but they were not used to build the model.

On MRI images we evaluated:

- The increased signal intensity of the inferior glenohumeral ligament on fat-saturated T2-weighted sequences;
- Axillary pouch thickening over 4 mm;
- Thickening of the coracohumeral ligament and capsule at the rotator cuff interval;
- Obliteration of the triangular fat pad inferior to the coracohumeral ligament;
- Poor capsular distension;
- Synovial hypertrophy;
- Tissue scarring at the rotator interval.

MRI dataset was composed of: Coronal STIR, Sagittal STIR, Sagittal T2, Coronal T1, Axial PD FS. All the sequences were acquired with thickness of 3,5 mm and FOV 160-180. Thus, based on this criterion, patients were categorized as either “capsulitis” or “non-capsulitis”, i.e. affected/not affected by AC disease.

For this retrospective study, we did not perform a Region of Interest (ROI) analysis on the images; rather, we examined the entire slice. On average, 22 slices per patient were analyzed, covering both coronal and sagittal acquisitions.

#### Methodology to automatically diagnose adhesive capsulitis

**Figure 1:**
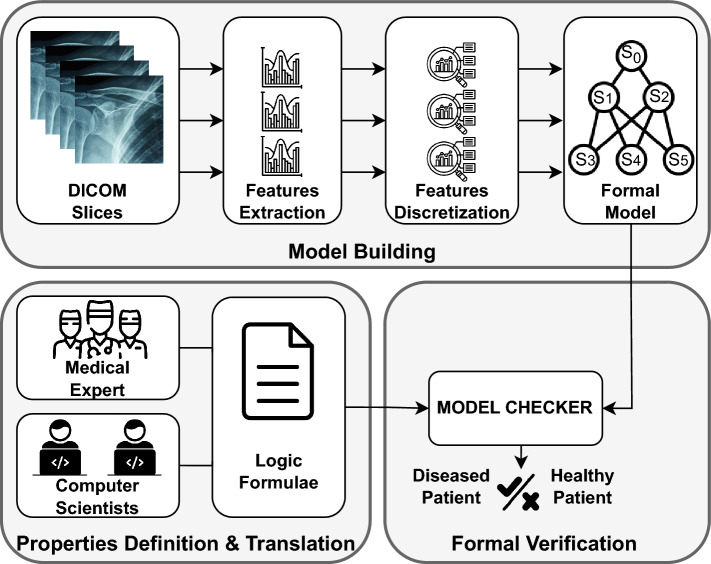
Workflow of our methodology.

We define a Model Checking-based methodology to diagnose AC using medical images. Figure 1 shows the workflow of our methodology consisting of three stages: (i) Model Building, (ii) Properties Definition & Translation, and (iii) Formal Verification. In the following, we provide a detailed overview of each stage in our methodology describing the steps linked to each respective stage.

*Feature extraction.* To build the formal model of a patient we need to collect radiomics features from a DICOM image. To do so, we use PyRadiomics^25^, an open-source Python package, to extract radiomics features from medical imaging. It aligns with the directives established by the Image Biomarker Standardization Initiative (IBSI)^26^ and facilitates the processing and extraction of radiomics features from medical imaging data using a wide range of engineered formulas and algorithms^26^. The available features are divided into six primary groups:

- *First-order features (FIRST).* These describe the distribution of voxel intensities within the considered image region. Within this group, one can distinguish features based on intensity and features based on the histogram, in total this group contains 19 different features;
- *Gray-level co-occurrence matrix features (GLCM). *These are part of textural features and provide spatial information about the distribution of gray levels. Specifically, they represent the number of times a combination of two levels occurs in two pixels in the image separated by a distance of $\delta$ pixels along an angle $\alpha$. It groups 24 different features;
- *Gray-level dependence matrix features (GLDM).* Gray-level dependence is defined as the number of connected voxels within the distance $\delta$ that depend on the central voxel. This group contains 14 features;
- *Gray-level run length matrix features (GLRLM). *Sequences are defined as the length, in the number of pixels, of consecutive pixels with the same gray value, in total it collects 16 features;
- *Gray-level size zone matrix features (GLSZM). *A zone is defined as the number of connected voxels that share the same gray intensity, in total this group contains 16 different features;
- *Neighborhood gray-tone difference matrix features (NGTDM). *It quantifies the difference between a gray-level value and the mean value of neighboring voxels. It contains 5 different features.

Initially, all the features of each class were computed. In total, we have 94 features for each patient for each sequence (coronal and sagittal). Due to the high number of features for each patient, we decided to perform Feature Selection to list all significant features not redundant. Successively, we carried out Feature Selection, analyzing radiomics features through Weka^27^, which is an open-source software developed in New Zealand under the General Public License. This reduction process yielded 30 features, categorized into the six classes defined in the radiomic standards. Feature Selection and the Model Building Step will be solved in single-class mode, i.e. features are studied by Feature Class and no connections between different classes are analyzed. Tables 1 and 2 list all features for each class involved in our experimentation.

**Table 1:** Most relevant *features* for FIRST, GLCM and GLDM classes.

FIRST	GLCM	GLDM
Energy	Cluster prominence	Dependence variance
Median	Contrast	Gray level non uniformity
Minimum	Difference average	Gray level variance
Total energy	Difference variance	High gray level emphasis
Variance	Id	Large dependence low gray level emphasis

**Table 2:** Most relevant *features* for GLRLM, GLSZM and SHAPE classes.

GLRLM	GLSZM	SHAPE
Gray level non uniformity	Gray level non uniformity	Flatness
Gray level variance	Large area emphasis	Least axis length
High gray level run emphasis	Large area high gray level emphasis	Mesh volume
Long run high gray level emphasis	Large area low gray level emphasis	Surface area
Run length non uniformity	Zone variance	Voxel volume

*Model building.* Before creating the formal model, we need to discretize the features. To do so, we used three levels of values: low *L*, medium *M*, and high *H*. Then, the discretized values were used to build the formal model. Basically, we model each slice of the DICOM image as a process that combines all relevant features. At the end of these steps, the formal model representing the patient is built.

Our formal model mimics how extracted features appear in each slice of a given medical exam. More in detail, using the Calculus of Communicating System (CCS)^28^, we can write models in which there are as many processes as there are radiological examination slices. In each process, there is a discretized level for each feature. An example of the CCS formal model is provided in Table 3. The operator nil determines the termination of the model. Also present in each process is the $+$ operator with which all alternating combinations of features are concatenated. If between two features F1, and F2, the process is F1.F2, the other combination will be F2.F1. Thus interleaving between features is implemented, i.e. their simultaneous execution. This means that the order in which features appear in a process does not matter.

To check whether a formal model of a patient exposes AC we need to define formal properties able to identify this disease over the image features.

**Table 3:** Example of CCS model of a patient.

proc P1	=	H_variance.H_energy.H_median.H_minimum.H_totalenergy.P2+ .
proc P2	=	H_variance.H_energy.H_median.H_minimum.H_totalenergy.P3+ .
proc P3	=	H_variance.M_energy.H_median.H_minimum.M_totalenergy.P4+ .
proc P4	=	H_variance.M_energy.H_median.H_minimum.M_totalenergy.P5+ .
proc P5	=	H_variance.L_energy.H_median.H_minimum.L_totalenergy.P6+ .
proc P6	=	H_variance.L_energy.H_median.H_minimum.L_totalenergy.P7+ .
proc P7	=	H_variance.L_energy.H_median.H_minimum.L_totalenergy.P8+ .
proc P8	=	H_variance.L_energy.H_median.H_minimum.L_totalenergy.P9+ .
proc P9	=	H_variance.L_energy.H_median.H_minimum.L_totalenergy.P10+ .
proc P10	=	H_variance.L_energy.H_median.H_minimum.L_totalenergy.P11+ .
proc P11	=	H_variance.L_energy.H_median.H_minimum.L_totalenergy.P12+ .
proc P12	=	H_variance.L_energy.H_median.H_minimum.L_totalenergy.P13+ .
proc P13	=	H_variance.L_energy.H_median.H_minimum.L_totalenergy.P14+ .
proc P14	=	H_variance.M_energy.H_median.H_minimum.M_totalenergy.P15+ .
proc P15	=	H_variance.H_energy.H_median.H_minimum.H_totalenergy.P16+ .
proc P16	=	H_variance.H_energy.H_median.H_minimum.H_totalenergy.P17+ .
proc P17	=	H_variance.H_energy.H_median.H_minimum.H_totalenergy.P18+ .
proc P18	=	nil

*Properties definition & translation.* To define formal properties identifying AC, we ask radiologists to select patients with evident disease symptoms. Starting from this subgroup of **6** patients, Formal Methods experts, and radiologists defined possible composition of features and discretization level eligible to identify the disease. Then, these compositions are translated into temporal logic formulae which aim to merge commonalities among patients in identical health states enhancing the visibility of their health condition and highlighting patterns indicating healthiness/disease.

*Formal verification.* The last stage of our methodology involves formal verification. To do so, a Model Checker tool is used to automatically verify formal properties against the patient models. This tool is able to explore the state space and verify the properties at each state. The Model Checker takes as input both the formal model and the properties. Following an assessment of whether the model fulfills the given property or not, it yields a binary truth value as its output: TRUE if the model meets the property, FALSE if it does not. In our specific case, if the Model Checker returns TRUE it means that the patient under analysis is affected by AC. Instead, an output equal to FALSE indicates that the patient does not present disease.

##### Participants’ selection and demographics

Given the study context, selecting a broader range of participants would not have been feasible, since our aim was to involve professionals with sufficient expertise in MSK. Hence, we selected participants through a convenience sampling process, among radiologists experienced with MSK. More in detail, we extended invitations to a total of 11 professional radiologists asking them to participate in the diagnostic task. We sent them an invitation message explaining the goals of our study and clarifying that (i) the participation in the experimentation is voluntary, (ii) personal data will be treated as strictly confidential, (iii) the approximate time to categorize patients was estimated to be about 20 min and a participant can withdraw at any time. The survey has been administered through e-mails. The whole process of participants’ selection, invitation, and collecting responses took about 1 month.

In the end, we gathered 11 responses as each selected participant completed the diagnostic task. The MSK radiology experience of respondents varies as follows:

- 1 radiologist with 20 years of experience in musculoskeletal radiology;
- 3 specialists in radiology with an average of 5–7 years of experience in MSK;
- 3 specialists in radiology with an average of 2 years of experience in MSK;
- 4 radiology residents with no years of experience in MSK.

For the sake of ease, we grouped participants into 3 levels of experience: 4 of the participants had a* Beginner Level* (Radiology Residents), 3 an *Intermediate Level* (Early Professionals), and 4 an* Expert Level* (Professionals Radiologists). Thus, to answer RQ$_2$ we involve three groups of radiologists categorized by their experience. Participants were asked to determine, based on anonymized MRI images complete of both coronal and sagittal planes, whether patients were affected by AC or not.

##### Analysis methodology

To address RQ$_1$, we report results achieved by our methodology on the dataset described in the Dataset Section. It should be underlined that, for each patient, we build a formal model considering both the two different orientations of the imaging plane, i.e., sagittal and coronal sequences. In particular, the model creation requires on average 0.11 seconds per patients having about 22 slices in its medical examination. Thus, for each patient, we extract and select features from both sequences to build the formal patient model as described in Model Building step.

Then, according to Properties Definition & Translation step, we define formal properties able to catch the distinctive disease marks. We define properties for both sagittal and coronal sequences, i.e., we combine properties for both imaging planes with the logical “OR” operator, hence the overall result is true if at least one of the individual plans is true. Note that to analyze a patients, i.e., to verify the formal property on the patient model, our approach requires 2.62 seconds per patients having about 22 slices. The execution times were recorded using a machine equipped with 13$^{th}$ Gen Intel(R) Core(TM) i9-13900HX (2.20 GHz) processor and 32GB RAM. As operating system it was equipped with Windows 11.

To address RQ$_2$, we show and compare results achieved by our methodology with those collected with administration through the diagnostic task survey, and we discuss them.

## Results

In the following section, we show the results achieved for both RQs.

### RQ$_1$: Can adhesive capsulitis be diagnosed using model checking and radiomics features?

**Table 4:** GLCM results for each MRI sequence or for their combination.

Property	Accuracy	Precision	Recall	Specificity
GLCM coronal	0.5636	0.9000	0.2812	0.9565
GLCM sagittal	0.6000	0.7777	0.4375	0.8261
GLCM sagittal + coronal	0.7455	0.8214	0.7188	0.7826

Confusion Matrix and performance indicators are computed for each of the defined properties. More in detail, the performance indicators used are Precision, Recall, Accuracy, and Specificity. Table 4 shows results for the best formula with the GLCM class. We tested it against three different models: (i) a formal model built using only MRI coronal plane, (ii) a formal model built using only MRI sagittal plane, and (iii) a formal model combining both coronal and sagittal planes. The formal single-plane model was designed to check if there was a plane showing more signs of AC than the other. As a result of the coronal plan, there is a high efficiency (i.e., 90%) in the classification of negative patients, but a low efficiency (i.e., 28%) in the recognition of AC patients. Conversely, the sagittal plan improves performance in recognizing sick patients from healthy ones but there is also an increase in false positive numbers. To sum up, using a single plan is not enough to detect the presence of AC disease. As depicted in Table 4, the best performance results from the combination of both two planes. Consider that, using both plans improves the completeness just compromising the Precision and Specificity values. However, this is still an acceptable result since we prioritize the method of detecting as many sick individuals as it can, even if it means misclassifying some healthy conditions in the process.

### RQ$_2$: To what extent does the proposed approach for adhesive capsulitis diagnosis perform in terms of accuracy when compared to traditional diagnostic modalities?

**Table 5:** Performance indicators of diagnostic task detailed by experience levels.

Experience	Accuracy	Precision	Recall	Specificity
Beginner level	0.5409	0.6053	0.5625	0.5109
Intermediate level	0.5334	0.6088	0.5834	0.4509
Expert level	0.7182	0.7680	0.7656	0.6522
**Our method**	**0.7455**	**0.8214**	**0.7188**	**0.7826**

Table 5 compares diagnostic results achieved by our method with those reached by radiologists. Note that, in Table 5 results of our method are the best performance achieved by our approach using both two planes, i.e., sagittal and coronal planes. For participants at the Beginner Level, i.e., radiology residents with low experience in MSK, performance metrics exhibit the lowest values compared with greater levels of experience. This result is not surprising since radiology residents are initiating their experience in the field. If we compare these results with those achieved by our method we can easily note that our solution outperforms them. Thus, it could be a suitable support tool to teach entry-level residents.

Regarding Intermediate Level radiologists, results show a slight improvement compared to the Beginner Level but remain inferior to the proficiency demonstrated by the experts and our method. In this case, a tool integrating our approach can be used as a recommendation system to aid intermediate level radiologists in improving their capabilities.

Finally, Expert Level radiologists achieved the best results nearly comparable to the performance reached by our approach, although our results are slightly better. It should be underlined that the radiologist with 20 years of experience demonstrated the highest overall performance, i.e., both Precision and Specificity equal to 1, a Recall of 0.9375, and an Accuracy equal to 0.9636. Therefore, our approach does not outperform a high experience in the field but it could remain a viable option for prioritizing more urgent cases.

## Discussion

In our research, we specifically focus on a group of patients, without prior knowledge of other comorbidity information, to accurately diagnose the presence of AC. Our methodology is designed to operate automatically, utilizing a single ROI created through a threshold-based method. The primary objective of our study is to effectively identify radiological features related to capsulitis. To the best of our knowledge, this is the first study concerning the automated diagnosis of AC in few minutes.

Our model achieved the best global accuracy in recognizing AC (74,5%), even better than expert radiologists. It can be speculated that expert general radiologists achieved a higher sensitivity and specificity, but, actually, they are used to overdiagnose AC findings. Overdiagnosis was not an issue for MSK specialized radiologists who achieved the best diagnostic performance.

Our results are encouraging because our model was not created for MSK radiologists but for general radiologists, who could benefit from a second opinion in few minutes. Concerning radiological and radiomics feature our model achieved the best accuracy analyzing both the sagittal phase, and the coronal phase. Comparing the results of automatic classification with those performed by medical specialists, the results of the tool are better in terms of false positives. The designed methodology succeeds in keeping the threshold of unhealthy people recognised, while lowering the number of healthy patients who are erroneously misdiagnosed by the possible presence of the disease. These results are possible thanks to the features of the GLCM class, which studies spatial correlations of pairs of pixels. Compared to other classes, these features manage to be more significant in the classification of the disease. The reported results are comparable to the ones found in literature, where the sensitivity of MSK radiologists is 0.84, with an high agreement when there is hyperintensity of the axillary recess. However the sensitivity related to other radiological features such as axillary recess CHL thickness $\ge$ 2 mm or axillary recess capsule in axillary recess $\ge$ 4 mm is about 0.50.

Concerning GLCM and Radiomics in the shoulder medical field, Triveni et al. attempted image Feature Extraction for the analysis of shoulder pain^29^. Please note that shoulder pain is not necessarily a Frozen Shoulder, but this is the first result of the efficacy of GLCM features. They propose a region- and watershed-based segmentation method applied to MRI images to identify shoulder disorders. They compared the Feature Extraction and Deep Feature Extraction techniques for an in-depth analysis of shoulder disorders and the type of shoulder pain. GLCM was found to be very good at diagnosing in which area the pain occurs and what type of pain it is. However, they did not classify AC patients, but they establish which algorithm is more accurate for shoulder segmentation. While they use different types of features, we only based the study on the textural GLCM values and without the need for AI techniques.

Furthermore, the usefulness of this feature class is also mentioned by Scott et al.^30^. Specialists claim that a segmentation based on GLCM has generated effective results for tendons in ultrasound imaging of patients with shoulder pain. The difference in the observed values between healthy and tendinopathic tendons emerges as a potential instrument for objectively assessing damage in tendinopathy. In our study, we focus on AC instead of tendinopathy. However, the use of GLCM in MRI is analyzed, confirming that this class of features can be just as effective as in the case of ultrasound imaging.

In the existing literature, there is limited research addressing the utilization of Radiomics for the diagnosis of AC. Most of the studies primarily focus on establishing correlations between AC and shoulder pain among cancer patients. Notably, AC frequently manifests in cancer patients, often occurring during chemotherapy or following surgical procedures, such as those related to breast cancer. In a study conducted by Hayashi et al.^31^, a cohort of patients underwent^29^ FDG PET-CT scans, during which SUV parameters were measured within manually delineated ROIs on transaxial images. The radiotracer uptake analysis revealed that symptomatic patients exhibited higher SUVmax and mean SUV values compared to asymptomatic patients. This observation underscores the importance of considering AC as a significant factor in cancer patients, particularly in cases where there is heightened metabolic activity, as noted in previous research by Yang et al.^32^.

These imaging techniques are employed because CT scans are effective in identifying inflammatory diseases, enabling the detection of hypermetabolic activity in the shoulder joint through PET-CT scans when active inflammation is present. As a matter of fact, AC patterns have been characterized in a study by Kim et al.^33^, involving 21 patients, in which SUVs were assessed within four distinct ROIs, revealing four distinct patterns associated with AC. However, it is worth noting that a limitation of using PET-CT is that these patterns are not entirely unique, as other medical conditions can sometimes exhibit similar radiographic findings, as discussed by Salem et al.^34^.

The diagnosis of adhesive capsulitis have a role in the management of the disease. Currently the treatment of AC is dedicated to people affected by severe symptoms, clinically diagnosed without imaging. MRI helps clinicians to identify all the different grade of disease to treat them. At the same time overdiagnosis of AC can cause overtreatment for shoulder pain. For these reasons MRI and US imaging are mandatory and artificial intelligence can help to recognize and correctly detect this pathology in non-referral center. Our study has some limitations. The retrospective nature of the study can create bias in patients selection, and the MRI protocols can be updated. The sample size is limited, but it was necessary to ensure an accurate clinical diagnosis. Further studies aim to validate the model in a prospective cohort in clinical practise.

## Conclusion

In this paper, we aimed to develop a Model Checking-based method to automatically diagnose Adhesive Capsulitis of the Shoulder (“Frozen Shoulder”) through medical imaging exams, i.e., MRIs. We assessed the presence of ACS in patients through the evaluation of their MRI coronal and sagittal planes, considering both a single plane per time or their union. Then, we assessed the performance of our method by surveying 11 radiologist professionals to compare their diagnosis results with those achieved by our method. Overall, the diagnostic results achieved by our method are slightly better than those of the surveyed professionals.

To the best of our knowledge, we are the first in this field to provide a technique for automatically classifying ACS (“Frozen Shoulder”). Furthermore, the use of Radiomics and Model Checking technique can assert the effectiveness of automatic tools that can assist radiology professionals in an early diagnosis of Adhesive Capsulitis.

## Data Availability

The dataset and the informed consent has been obtained following the guidelines of the ethics committee (prot. no. 27789 of 12.06.2024). The dataset used and analysed during this study are available from the corresponding author upon reasonable request.

## Abbreviations

ACS or AC: Adhesive capsulitis of the shoulder
MRI: Magnetic resonance imaging
MSK: musculoskeletal experts
AI: Artificial intelligence
CT: Computed tomography
PET: Positron emission tomography
ROI: Region of interest
LTS: Labelled transition system
RQ: Research question
DICOM: Digital Imaging and Comunications in Medicine
IBSI: Image biomarker standardisation initiative
CCS: Calculus of communicating system
